# Supplementary material for: Sub-optical-cycle light-matter energy transfer in molecular vibrational spectroscopy
Source: Nat Commun. 2022 Oct 6;13:5897. doi: 10.1038/s41467-022-33477-5 (PMC9537163; doi:10.1038/s41467-022-33477-5)
Supplement: Supplementary file 1 — Supplementary Information [file 41467_2022_33477_MOESM1_ESM.pdf]

Supplementary Information

Sub-optical-cycle light-matter energy transfer in molecular vibrational spectroscopy

Martin T. Peschel[1,*], Maximilian Högner[2,3,*], Theresa Buberl[2,3,*], Daniel Keefer[1,4], Regina de Vivie-Riedle[1✉], Ioachim Pupeza[2,3✉]

1. Ludwig-Maximilians-Universität München, Butenandtstraße 5-13, 81377 Munich, Germany

2. Max-Planck-Institut für Quantenoptik, Hans-Kopfermann-Straße 1,85748 Garching, Germany

3. Ludwig-Maximilians-Universität München, Am Coulombwall 1, 85748 Garching, Germany

4. Department of Chemistry, University of California, Irvine, CA 92697, USA

** these authors contributed equally*

✉ *corresponding authors, e-mail: regina.de_vivie@cup.uni-muenchen.de, ioachim.pupeza@mpq.mpg.de*

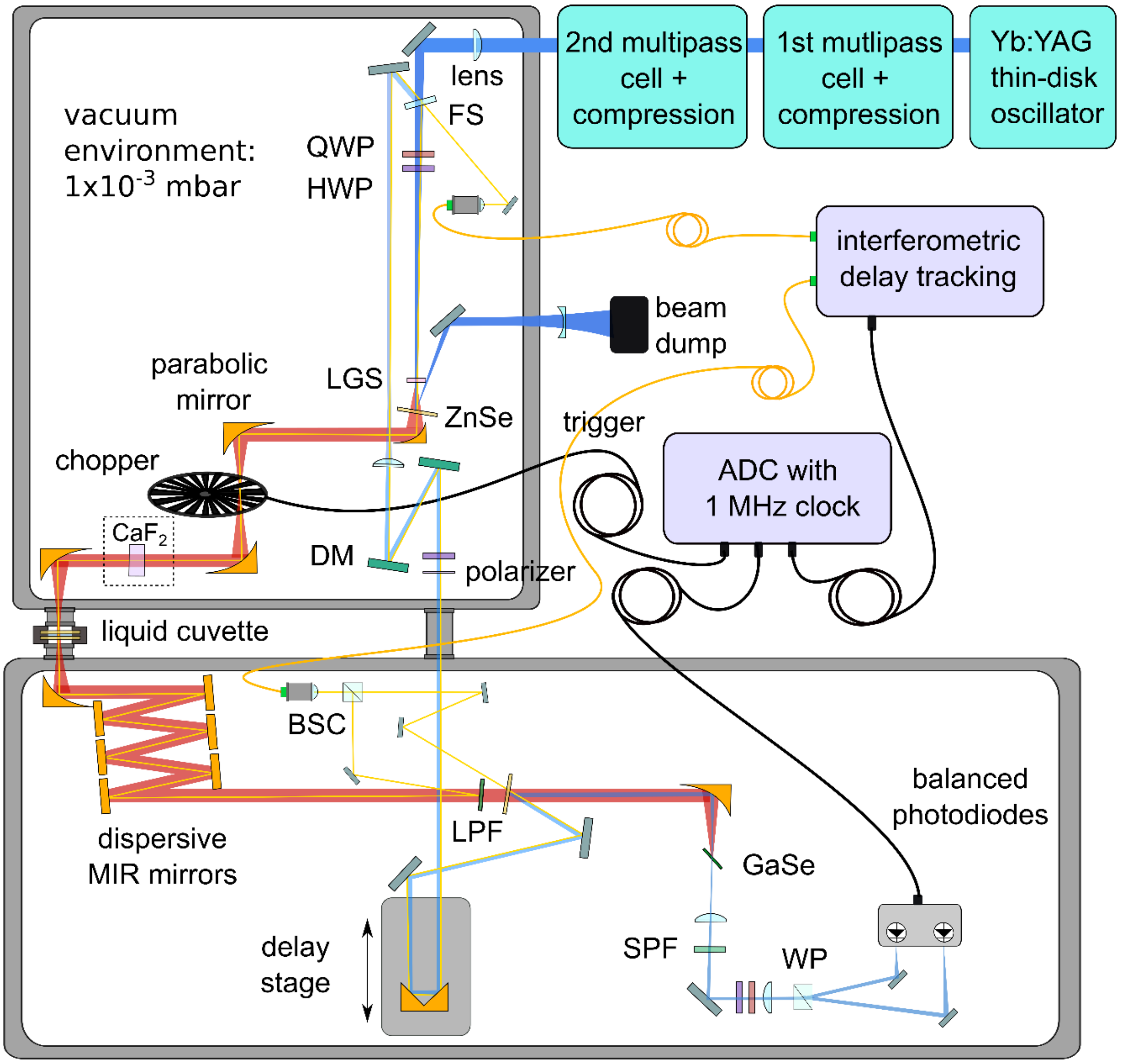

Supplementary Figure 1: Experimental setup. For details, see Methods in the main text, subsection "Field-resolved spectroscopy". FS: fused silica, QWP: quarter-wave plate, HWP: half-wave plate, CW: continuous wave laser, LGS: $LiGS_2$ crystal, ZnSe: dichroic mirror on ZnSe substrate, DM: dispersive mirror, ADC: analog-to-digital converter, CaF_2: uncoated CaF_2 window, placed under normal incidence, BSC: beam splitter cube, LPF: long-pass filter, GaSe: GaSe crystal, SPF: 950-nm short-pass filer, WP: Wollaston prism.

Supplementary Table 1: Coordinates of $DMSO_2$. Optimized using M06-2X/6-31g* in PCM(water).

Element	X(Å)	Y(Å)	Z(Å)
S	0.000001	0.170163	0.000013
O	-0.000147	0.931109	1.259878
O	0.000072	0.931253	-1.259795
C	1.411790	-0.914460	0.000040
H	1.383754	-1.527002	0.901244
H	1.383567	-1.527199	-0.901025
H	2.291847	-0.269406	-0.000140
C	-1.411730	-0.914541	-0.000128
H	-1.383587	-1.527033	-0.901362
H	-1.383519	-1.527324	0.900908
H	-2.291831	-0.269545	0.000028

Supplementary Table 2: Normalized displacement vectors of the symmetric and asymmetric stretching vibration of $DMSO_2$. Calculated by harmonic frequency analysis using M06-2X/6-31g* in PCM(water), visualised in Figure 2b of the main text.

Element	Mode 14 (symmetric stretch)			Mode 15 (asymmetric stretch)		
	X	Y	Z	X	Y	Z
S	-0.00	0.25	-0.00	-0.00	0.00	0.43
O	0.00	-0.18	-0.27	0.00	-0.23	-0.40
O	-0.00	-0.18	0.27	0.00	0.23	-0.40
C	-0.03	-0.09	0.00	-0.00	-0.00	-0.07
H	0.37	-0.04	0.05	-0.06	0.20	0.08
H	0.37	-0.04	-0.05	0.06	-0.20	0.08
H	-0.21	0.18	0.00	0.00	-0.00	0.29
C	0.03	-0.09	0.00	0.00	-0.00	-0.07
H	-0.37	-0.04	-0.05	-0.06	-0.20	0.08
H	-0.37	-0.04	0.05	0.06	0.20	0.08
H	0.21	0.18	-0.00	0.00	-0.00	0.29

Supplementary Table 3: Harmonic vibrational frequencies and IR intensities of $DMSO_2$. Calculated by harmonic frequency analysis using M06-2X/6-31g* in PCM(water).

Mode #	Wavenumber (cm^{-1})	IR Intensity (km mol^{-1})
1	170.5	0.00
2	249.8	1.21
3	284.5	4.73
4	290.8	0.00
5	358.5	2.95
6	439.0	83.23
7	474.9	63.58
8	701.2	7.62
9	775.9	50.31
10	951.7	0.00
11	967.0	94.31
12	1018.2	1.61
13	1033.8	1.45
14	1135.5	326.63
15	1272.0	537.71
16	1353.4	19.14
17	1378.2	21.33
18	1440.9	11.36
19	1441.9	0.00
20	1453.4	11.35
21	1467.7	16.78
22	3097.1	1.08
23	3103.1	0.08
24	3210.1	2.10
25	3213.3	1.15
26	3215.9	0.00
27	3225.3	1.21

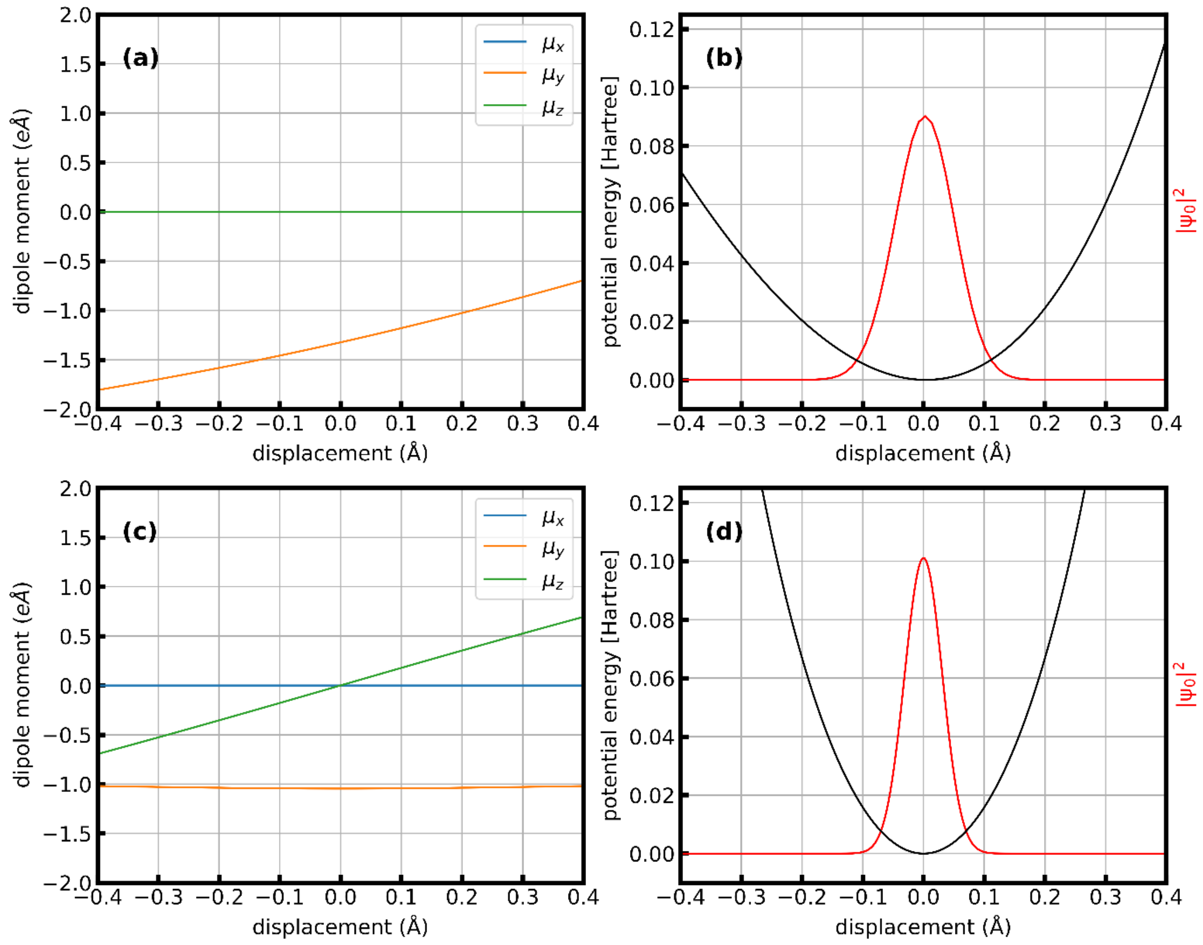

Supplementary Figure 2: Dipole moments, potential energy surfaces and vibrational ground state probability densities for the symmetric and asymmetric stretching vibration of $DMSO_2$. (a) Dipole Moments of $DMSO_2$ when displaced along normal mode 14 (asymmetric stretch). (b) Potential energy and ground state wavefunction in normal mode 14 (asymmetric stretch). (c) Dipole Moments of $DMSO_2$ when displaced along normal mode 15 (symmetric stretch). (d) Potential energy and ground state wavefunction in normal mode 15 (symmetric stretch).

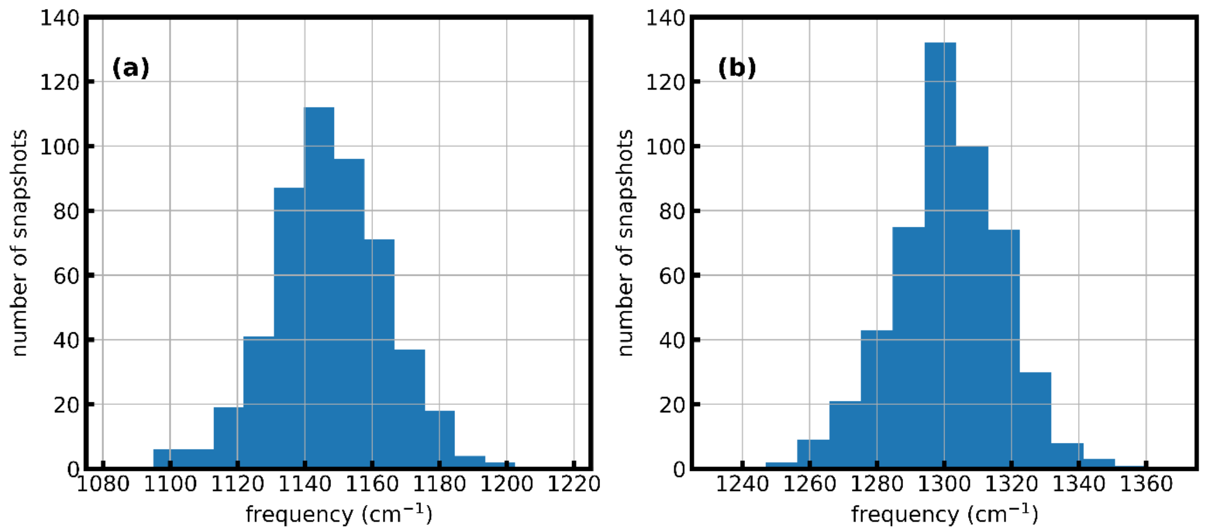

Supplementary Figure 3: Static frequency distributions of $DMSO_2$ water clusters. (a) mode 14 (symmetric stretch) and (b) mode 15 (asymmetric stretch). $DMSO_2$ water clusters were obtained from snapshots of classical molecular dynamics simulations as described in the Methods section of the main text. Frequencies were evaluated using the anharmonic approach based on M06-2X/6-31g*/PCM(water) single point calculations.

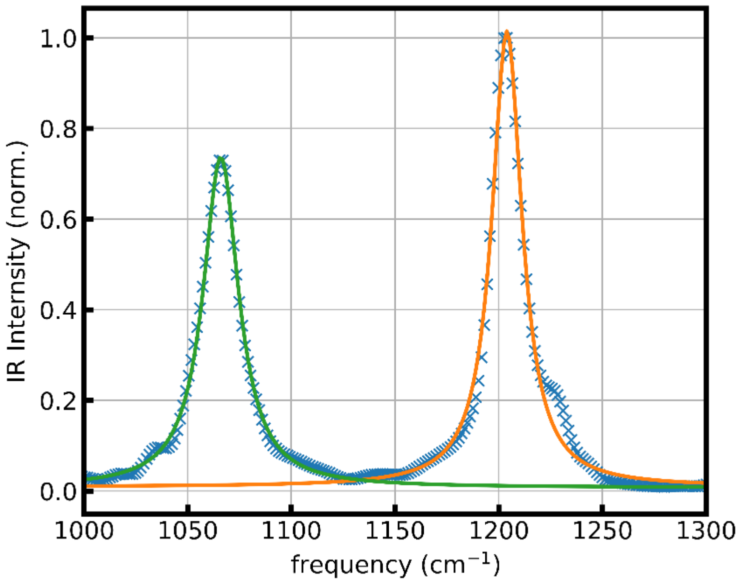

Supplementary Figure 4: Estimation of dephasing rates from molecular dynamics simulation. Fourier-transformed dipole autocorrelation function from *ab initio* molecular dynamics (blue crosses), fit of Lorentzian line shape to the absorption band corresponding to the symmetric stretch (green) and the asymmetric stretch (orange). From the fit, a FWHM Γ/π was determined as 21.0 cm^{-1} for the symmetric stretch and 17.8 cm^{-1} for the asymmetric stretch, giving an averaged estimate of FWHM = 19.4 cm^{-1}.

Supplementary Note 1

Propagation through the medium

1D Propagation of the electric field $\mathcal{E}(\omega, z)$ in a linear dielectric medium is described by the electromagnetic wave equation

$$(\mu_0^{-1}\epsilon_0^{-1}n^{-2}(\omega)\,\partial_z^2 + \omega^2)\mathcal{E}(\omega, z) = 0, \quad (1)$$

which follows without approximations from the Maxwell equations in linear media. Its solution is

$$\mathcal{E}(\omega, z) = \mathcal{E}(\omega, 0)\exp\left(-in(\omega)\frac{\omega}{c}z\right), \quad (2)$$

with the complex refractive index $n(\omega) = \sqrt{1 + \chi^{(1)}(\omega)}$, where the complex susceptibility $\chi^{(1)}(\omega)$ follows from the complex molecular polarizabilities $\alpha(\omega)$ and number densities ρ_N of the constituents of the water-$DMSO_2$ mixture via the low-concentration approximation of the Clausius-Mosotti relation:[1]

$$\chi^{(1)}(\omega) = \sum_{i\in\{H_2O, DMSO_2\}} \rho_{N,i}\alpha_i(\omega) \quad (3)$$

The complex molecular polarizability of $DMSO_2$, in turn, follows from the Lorentz parameters of the vibrational transitions:

$$\alpha_{DMSO_2}(\omega) = \frac{2}{3}\frac{\epsilon_0}{\hbar}\sum_k \frac{|\mu_k|^2\omega_k}{\omega_k^2 - (\omega + i\Gamma)^2}, \quad (4)$$

where k enumerates the transitions, and μ_k, ω_k and Γ are the transition dipole moments, transition angular frequencies, and relaxation constant, respectively.

Supplementary Note 2

Role of the reference measurement

For small concentrations, one may approximate $n(\omega) \approx 1 + \chi^{(1)}(\omega)/2$ and therefore

$$\mathcal{E}(\omega, z) \approx \mathcal{E}(\omega, 0)\exp\left(-i\left(1 + \chi^{(1)}(\omega)/2\right)\frac{\omega}{c}z\right) =$$

$$= \mathcal{E}(\omega, 0)\exp\left(-i\frac{\omega}{c}z\right)H_{H_20}(\omega)H_{DMSO_2}(\omega), \quad (5)$$

with the transfer functions

$$H_{DMSO_2}(\omega) = \exp\left(-i\,\rho_{N,DMSO_2}\alpha_{DMSO_2}(\omega)/2\,\frac{\omega}{c}z\right) \quad (6)$$

and

$$H_{H_20}(\omega) = \exp\left(-i\,\rho_{N,H_20}\alpha_{H_20}(\omega)/2\,\frac{\omega}{c}z\right). \quad (7)$$

We can rearrange like

$$\mathcal{E}(\omega, z) \approx \underbrace{\mathcal{E}(\omega, 0)H_{H_20}(\omega)\exp\left(-i\frac{\omega}{c}z\right)}_{=:\,\mathcal{E}\prime(\omega,0)} \times \exp\left(-i\frac{\omega}{c}0\right)H_{DMSO_2}(\omega), \quad (8)$$

which is formally equivalent to the propagation through an infinitesimally thin slice of $DMSO_2$ molecules (with the number of molecules per cm^2 kept constant as compared to the mixed $DMSO_2$

and water sample), with an initial field $\mathcal{E}'(\omega, 0)$ in place of $\mathcal{E}(\omega, 0)$. This initial field is exactly what is measured in the reference measurement, and the field at the end of the $DMSO_2$ sample $\mathcal{E}(\omega, z)$ is identical to the sample measurement:

$$\mathcal{E}'(\omega, 0) = \mathcal{E}_{\mathrm{ref}}(\omega) \tag{9}$$

$$\mathcal{E}(\omega, z) = \mathcal{E}_{\mathrm{sam}}(\omega) \tag{10}$$

Consequently, the quantity defined in Eq. (1) of the main text,

$$\mathrm{CET}(t) = c\epsilon_0 \int_{t_0}^{t} \left(\mathcal{E}_{\mathrm{ref}}^2(t') - \mathcal{E}_{\mathrm{sam}}^2(t')\right) \mathrm{d}t', \tag{11}$$

describes the coherent energy transfer between this hypothetical, infinitesimally thin $DMSO_2$ sample and the pulse measured in the reference measurement.

Supplementary Note 3

Absorbed and reemitted energy in the limit of an impulsive excitation

The final level of the CET, i.e., the energy that remains in the $DMSO_2$ sample (see *Role of the reference measurement*) is the difference between the energy in the sample and the reference pulse:

$$E_{\mathrm{final}} = \int [\overline{S}_{\mathrm{ref}}(\omega) - \overline{S}_{\mathrm{sam}}(\omega)]\, \mathrm{d}\omega, \tag{12}$$

where $\overline{S}_{\mathrm{ref}}(\omega) \propto |\mathcal{E}_{\mathrm{ref}}(\omega)|^2$, $\overline{S}_{\mathrm{sam}}(\omega) \propto |\mathcal{E}_{\mathrm{sam}}(\omega)|^2$ are the energy spectral densities of the reference and sample pulse, respectively.

The reemitted energy, i.e., the energy that is emitted by the $DMSO_2$ sample after the impulsive excitation, can be obtained as follows:

$$\mathcal{E}_{\mathrm{sam}}(\omega) = \mathcal{E}_{\mathrm{ref}}(\omega) H_{\mathrm{DMSO_2}}(\omega) \tag{13}$$

$$\Rightarrow \mathcal{E}_{\mathrm{sam}}(t) = \mathcal{E}_{\mathrm{ref}}(t) * H_{\mathrm{DMSO_2}}(t) = \mathcal{E}_{\mathrm{ref}}(t) * \left(H_{\mathrm{DMSO_2}}(t) - \delta(t)\right) + \mathcal{E}_{\mathrm{ref}}(t). \tag{14}$$

The second term is non-zero only during the time window of the exciting pulse. Therefore, we conclude that in the limit of a short pulse, the first term describes the reemitted radiation:

$$\mathcal{E}_{\mathrm{reem.}}(t) := \mathcal{E}_{\mathrm{ref}}(t) * \left(H_{\mathrm{DMSO_2}}(t) - \delta(t)\right) \tag{15}$$

Using the Plancherel theorem, we get the reemitted energy

$$E_{\mathrm{reem.}} = \int C |\mathcal{E}_{\mathrm{reem.}}(t)|^2\, \mathrm{d}t = \int C \left|\mathcal{E}_{\mathrm{ref}}(t) * \left(H_{\mathrm{DMSO_2}}(t) - \delta(t)\right)\right|^2 \mathrm{d}t =$$

$$= \int C \left|\mathcal{E}_{\mathrm{ref}}(\omega)\left(H_{\mathrm{DMSO_2}}(\omega) - 1\right)\right|^2 \mathrm{d}\omega = \int \overline{S}_{\mathrm{ref}}(\omega) \left|H_{\mathrm{DMSO_2}}(\omega) - 1\right|^2 \mathrm{d}\omega. \tag{16}$$

The energy that is temporally absorbed by the $DMSO_2$ sample (corresponding to the value of the CET just after the impulsive excitation) is just the sum of between the energy that remains in the sample and the reemitted energy:

$$E_{\mathrm{abs.}} = E_{\mathrm{final}} + E_{\mathrm{reem.}} \tag{17}$$

Supplementary Note 4

Scaling behaviour of the absorbed and reemitted energy with concentration

For small concentrations, the maximum absorbed energy scales linearly with the concentration (Fig. 4e of the main text). In contrast, the coherent re-emission scales quadratically with the concentration. This can be explained by a simple examination of the terms contributing to the electric field: For small concentration (see Supplementary Figure 5), the change of the electric field is proportional to the mass concentration β of the $DMSO_2$ molecules:

$$\mathcal{E}_{\text{sam}}(\omega) - \mathcal{E}_{\text{ref}}(\omega) = \mathcal{E}(\omega, z) - \mathcal{E}'(\omega, 0) = \mathcal{E}'(\omega, 0)\big(H_{\text{DMSO}_2}(\omega) - 1\big) \approx$$

$$\approx \mathcal{E}'(\omega, 0)\, \frac{-i\, \rho_{N,\text{DMSO}_2} \alpha_{\text{DMSO}_2}(\omega)}{2} \frac{\omega}{c} z \propto \rho_{N,\text{DMSO}_2} \propto \beta \qquad (18)$$

This proportionality can be written in the time domain, where the proportionality constant *C(t)* contains the molecular response:

$$\mathcal{E}_{\text{sam}}(t) - \mathcal{E}_{\text{ref}}(t) = \beta \cdot C(t) \quad \Rightarrow \quad \mathcal{E}_{\text{sam}}(t) = \mathcal{E}_{\text{ref}}(t) + \beta \cdot C(t) \qquad (19)$$

Therefore, the integrand of the CET reads:

$$I_{\text{ref}}^{\text{inst}}(\text{t}) - I_{\text{sam}}^{\text{inst}}(t) = \mathcal{E}_{\text{ref}}^2(t) - \big(\mathcal{E}_{\text{ref}}(t) + \beta \cdot C(t)\big)^2 = -\beta^2 \cdot C^2(t) - 2\beta \cdot \mathcal{E}_{\text{ref}}(t) C(t). \qquad (20)$$

Because $\Delta\mathcal{E}(t)$ is much smaller than $\mathcal{E}_{\text{ref}}(t)$, the second term containing $\mathcal{E}_{\text{ref}}(t)$ is dominant during the FCE, which explains the linear scaling of the maximum absorbed energy. After the FCE, $\mathcal{E}_{\text{ref}}(t)$ and therefore the entire second term is zero so that the first term, containing a quadratic scaling factor with concentration, becomes dominant. In other words, the electric field of the molecular response after the FCE scales linearly with the concentration and the emitted energy therefore scales quadratically. In contrast, interference with the impinging pulse during the FCE leads to a predominantly linear concentration scaling of the energy absorbed during the FCE.

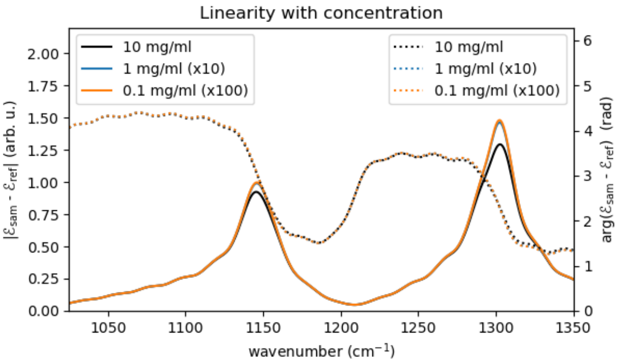

Supplementary Figure 5: Linearity with Concentration. Difference between the reference and sample field (solid: absolute value of its complex spectrum; dotted: spectral phase), for the $DMSO_2$ concentration in our experiment (10 mg/ml, black) and a concentration reduced by a factors of 10 and 100, respectively (blue and orange curves; scaled by the corresponding factors). For low concentrations (0.1 and 1 mg/ml), the difference is proportional to the concentration. For 10 mg/ml, the relative deviation from the linear behaviour due to saturation of the field impinging on the sample is below 14%.

Supplementary Note 5

Connection between CET(t) and vibrational coherence

When incoherent processes are neglected, our system is in a pure state characterized by a wavefunction $\Psi(t)$, with coefficients $\{c_l(t)\}$ for the first vibrational eigenstate of each vibrational mode l. The square of the absolute value of the coherence $\rho_{0k}(t)$ in one specific mode k can then be expressed as

$$|\rho_{0k}(t)|^2 = |c_0(t)^* c_k(t)|^2 = |c_k(t)|^2(1 - \sum_{l>0} |c_l(t)|^2) = |c_k(t)|^2 - \sum_{l>0} |c_k(t)|^2 |c_l(t)|^2 \quad (21)$$

With weak excitation, $|c_l(t)|^2 \ll 1$ for all $l > 0$ and the first term dominates

$$|\rho_{0k}(t)|^2 \approx |c_k(t)|^2. \quad (22)$$

$\mathrm{CET}(t)$ directly measures how much energy is removed from the excitation field at each point in time. By energy conservation, and when only a single vibrational transition per molecule is excited, $\mathrm{CET}(t)$ is proportional to the change in energy expectation value of one molecule undergoing that transition

$$\mathrm{CET}(t) \propto \hbar\omega_k \rho_{kk}(t) = \hbar\omega_k |c_k(t)|^2 \quad (23)$$

with the energy associated with the vibrational transition $\hbar\omega_k$. This shows the proportionality of $\mathrm{CET}(t)$ and $|\rho_{0k}(t)|^2$ under the given assumptions.

Supplementary Note 6

Simulation of vibrational coherences

As described in the main text, the vibrational coherences can be calculated using first-order perturbation theory as

$$\rho_{0k}^{(1)}(t) = \frac{i}{\hbar} \mu_{0k} \int_0^\infty \mathcal{E}(t - t_1) \mathrm{e}^{\mathrm{i}\omega_k t_1} \mathrm{e}^{-\Gamma_k t_1} \mathrm{d}t_1. \quad (24)$$

To generate the plots in figures 4a-d of the main text, the above equation was integrated numerically using MATLAB. We used the experimentally measured electric field of the reference pulses $\mathcal{E}(t)$ and the Lorentzian parameters μ_{0k}, ω_k and Γ_k of the *ab initio* model described in the main text. The resulting coherences $\rho_{0k}^{(1)}(t)$ transformed to a rotating frame by multiplying by $\mathrm{e}^{\mathrm{i}\omega_k t}$. For figures 4b-d of the main text, prior to the procedure described above, we Fourier-transformed $\mathcal{E}(t)$, removed all negative frequency components and reversed the transformation. This is equivalent to the rotating wave approximation.

Supplementary References

1. Van Rysselberghe, P. Remarks concerning the Clausius-Mossotti Law. *J. Phys. Chem.* **36**, 1152–1155 (1932).
